# A large dataset of labelled single tree point clouds, QSMs and tree graphs

**DOI:** 10.1038/s41597-025-06421-7

**Published:** 2025-12-17

**Authors:** Nils Griese, Martin Ritzert, Nils Nölke

**Affiliations:** 1 Department of Forest Inventory and Remote Sensing, University of Göttingen, Göttingen, Germany; 2 Institute of Computer Science and Campus Institute Data Science, University of Göttingen, Göttingen, Germany

**Keywords:** Forest ecology, Forestry

## Abstract

High-resolution data of individual trees are critical for advancing forest monitoring, inventory development, and ecological research. This dataset, BioDiv-3DTrees, comprises 4,952 individual tree point clouds of 19 species, captured using Terrestrial Laser Scanning (TLS) and Unmanned Aerial Vehicle Laser Scanning (ULS), along with 3,386 Quantitative Structure Models (QSMs) and graph representations of the 14 broadleafed species in the dataset. The trees were sampled across the three research areas of the Biodiversity Exploratories in Germany. Each tree is linked to an existing open-access forest inventory dataset, which includes species identity, diameter at breast height (DBH), and tree height. The dataset is suitable for various research applications, including biomass estimation, algorithm development, tree structure analysis, and data fusion with traditional inventory methods. All QSMs were generated using TreeQSM 2.4.1 and have been validated for tree height, diameter at breast height and crown projection area against their underlying point clouds to ensure consistency. The dataset provides a reliable and scalable resource for forest science and remote sensing communities.

## Background & Summary

Understanding and quantifying tree structures, such as tree volume or tree crown architecture, is essential for ecological studies, forest management, and environmental monitoring^1^. Advancements in methodologies such as Quantitative Structure Models (QSM), Terrestrial Laser Scanning (TLS) and Unmanned Aerial Laser Scanning (ULS) have revolutionized the analysis tree geometry and biomass, providing critical insights into forest growth and carbon storage^2–4^. These approaches offer detailed and accurate representations of tree structures, supporting applications ranging from sustainable forest management to climate change mitigation.

TLS, especially during leaf-off conditions, provides unparalleled accuracy in capturing the surfaces of stems and branches necessary for high-quality QSMs. Apart from QSMs, TLS data also enables standalone biomass estimation and analysis of tree concurrence^5–7^. The ongoing evolution of TLS technology has further increased its accessibility and application. Machine learning models now allow to segment individual trees from raw point clouds, broadening the potential for large-scale forest inventory applications and ecological research^8,9^.

Similarly, ULS has emerged as a transformative tool in forest research and management, providing cost-effective, large-scale laser scanning data of forest stands. Its ability to capture detailed data on forest canopies enhances the understanding of forest heterogeneity and tree crown competition, which are essential for sustainable management practices^10–12^. In addition to measuring tree height and crown projection area, ULS offers insights into vertical forest structure and canopy height profiles, both of which are critical for ecological studies^12,13^. Furthermore, ULS can enhance estimation of above-ground biomass (AGB), reducing the need for labor-intensive field measurements, and extending applicability to regions with limited ground-truth data^14^.

QSMs provide access to geometric and volumetric information about trees using high-resolution point clouds, such as those obtained with TLS. This is achieved by reconstructing the above-ground woody volume of a tree as a set of 3D cylinders. The resulting models can be used to analyze tree crown architecture and for extracting key parameters such as tree volume and height, or to classify tree species — pivotal variables for understanding forest dynamics and carbon storage^15^.

To our knowledge, few public datasets cover all described information sources. With *pytreedb* there is a dataset of 1,951 single trees of 24 tree species^16^. While *pytreedb* covers areas mostly in southern Germany, trees of northern Germany are not part of this dataset. Further, only for 77 trees there are TLS- and ULS-point clouds and forest inventory data available. The dataset *FOR-species20K* provides over 20,000 trees of 33 species, which were captured worldwide with multiple TLS, ULS, and Mobile Laser Scanning (MLS) devices^17^. However, both datasets do not provide a QSM representation of their trees. The dataset of Yazdi *et al*. provides 3,755 single tree point clouds, their corresponding QSMs, tree structure metrics and graph representations for urban trees^18^.

With BioDiv-3DTrees, we provide a high-quality dataset of TLS and ULS point clouds of 4,952 trees of 19 tree species, collected in leaf-off condition across multiple forest sides around Germany^19^. Species labels are provided for all trees in the dataset. The underlying open-access forest inventory data^20^ can be used to further increase the potential of our dataset. Additionally, we provide the corresponding QSM reconstructions for 3,386 broadleafed trees of 14 species, as well as their raw graph representation. This combination of high-quality TLS and ULS point clouds, QSM reconstructions and matched forest inventory data, renders this dataset particularly well-suited to training machine learning models for species classification, or for benchmarking 3D reconstruction algorithms^21,22^.

## Methods

### Study sites

**Fig. 1:**
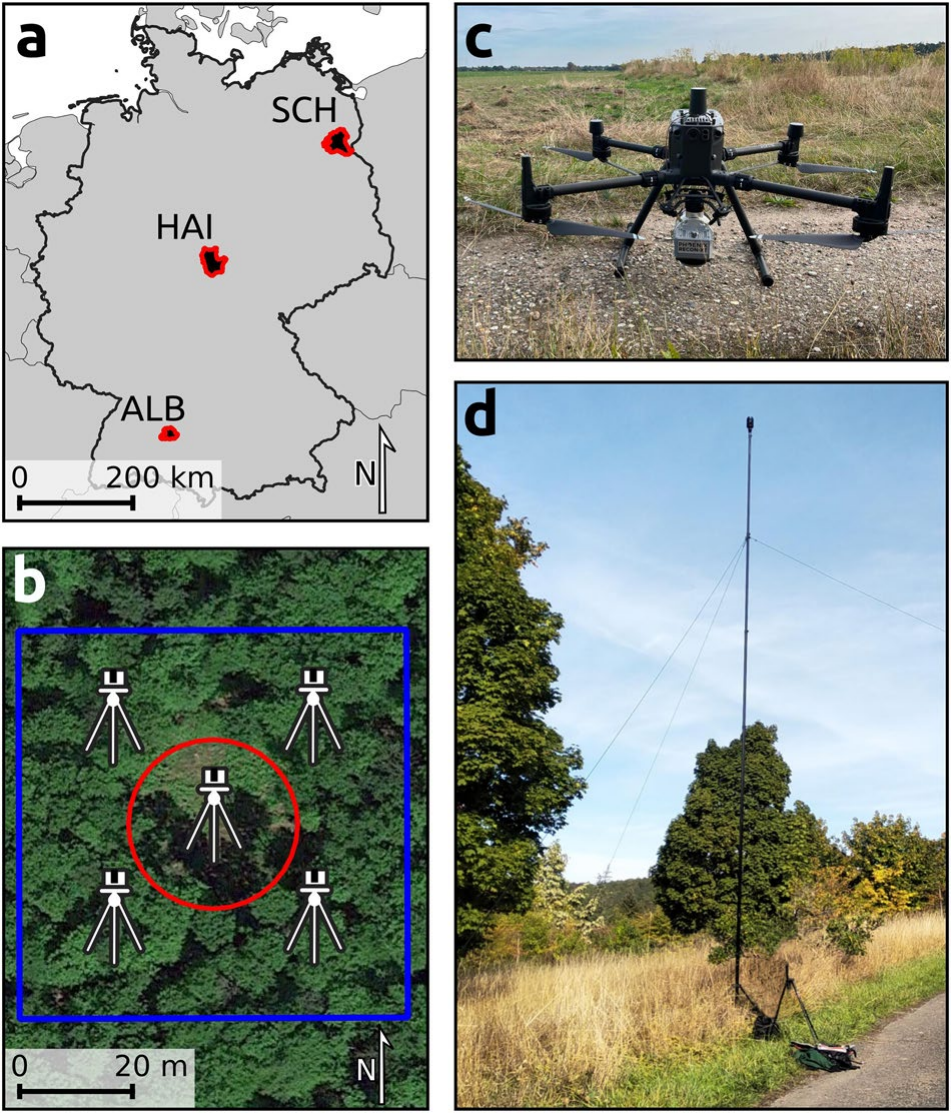
Overview of the data acquisition with the location of the three areas (**a**), the schematic of the on-plot scanner positions (**b**), the used drone setup (**c**), and the TLS setup (**d**). The blue square in B marks the boundaries of the plot, the red circle the extent of the created forest gap, and the five tripods mark the scanning positions per plot.

The data were collected in the three distinct regions of the Biodiversity Exploratories^23^ in Germany, which is a long-term research project to investigate the effects of varying land-use intensities on functional biodiversity (see Fig. 1). Schorfheide-Chorin (SCH) in the north-east of Germany is mainly covered by Scots pine (*Pinus sylvestris*), Sessile oak (*Quercus petraea)* and European beech (*Fagus sylvatica*). Hainich-Dün (HAI) in the center of Germany is covered by European beech and other hardwoods like European ash (*Fraxinus excelsior*). Schwäbische Alb (ALB), in the south-west of Germany is mainly covered in European beech and Norway spruce (*Pica abies*). Further information about the management type and species composition of the scanned forests can be found in the forest type classification dataset of the Biodiversity Exploratories^24^.

Experimental FOX-plots (FOrest gap eXperiment) were established in 29 forests across these regions in 2020 to study the impact of forest manipulations. Trees were removed within a 12.5–18.5 m radius around the plot centers. In each forest, two openings were created: deadwood was left behind in one (gap-deadwood plot, “GD”), while it was removed in the other (gap-only plot, “G”). This dataset is based on a scanning campaign of 27 of these forests and their G and GD subplots, resulting in a total of 54 scanned plots.

### Data acquisition

#### Terrestrial Laser Scanning (TLS)

We used a Leica BLK360 G1 terrestrial laser scanner, mounted on an extendable pole, to capture detailed three-dimensional tree structures across designated plots. The scanner achieves an accuracy of ±2 mm at 10 m distance, has a range of up to 60 m, and operates at a measurement rate of 360,000 points per second. Its field of view is 360° horizontally and 300° vertically, with the vertical field only limited by the scanner’s base. The beam divergence of the BLK 360 G1 is 0.68 mrad^25^.

Our scanning pattern consisted of one scan positioned at the gap center and four additional scans placed within the forest at the intercardinal directions (NE, SE, SW, NW) relative to the center point. At each scanning position, a scan at a height of 8 m and 2.2 m above ground was conducted. This configuration ensured comprehensive coverage of the forest plots and especially the upper tree crowns of the surrounding trees, improving scanning quality of crowns compared to traditional scanning heights at around 1.5–2 m without the additional scan at 8 m. The scanner was operated at its maximum resolution, resulting in dense point clouds with approximately 65 million points per scan.

#### Drone-Based LiDAR (ULS)

To complement the ground-based TLS data, drone-based LiDAR surveys were conducted by MapCad3D GmbH using a DJI Matrice 300 RTK equipped with a Phoenix LiDAR Systems Recon-XT scanner. Flights were executed at an altitude of 75 m above ground level and a flight speed of 6 m/s. The flight paths followed parallel lines to ensure uniform and efficient data collection. The resulting point clouds achieved a mean density of 604 points/m², enabling the generation of high-resolution three-dimensional representations of forest structures over larger spatial extents.

#### Data preprocessing

To ensure the reliability and consistency of the final tree segments, the preprocessing pipeline was designed with a focus on quality and alignment with the existing forest inventory. We adopted a conservative parameter selection strategy, guided by the principle that it is preferable to exclude a small portion of data if doing so increases the overall confidence in the remaining segments. This approach helped minimize mismatches and ensures that the final dataset consists of accurate and complete tree representations.

The TLS point clouds for each gap were co-registered using Leica’s Cyclone Register 360 + software (version 2023.0.2)^26^. The corresponding ULS point clouds were processed using Phoenix LiDAR’s SpatialExplorer software (version 7.0.8)^27^. To match the TLS point clouds with their respective ULS point clouds, we utilized CloudCompare (version 2.13)^28^ and its integrated Iterative Closest Point (ICP) algorithm. TLS and ULS point clouds were then merged while labeling each point’s source using the *PointSourceID* attribute of the used LAS files. After clipping the data to a circular area around the plot center with a radius of 50 m to reduce computation time for the following steps, we segmented the combined point clouds using TreeLearn^8^ with its default parameters, which operates directly on the point cloud. Disconnected point clusters within the single-tree segments were removed using histogram-based outlier detection, as implemented in the R package *bigutilsr* (version 0.3.4)^29^, where we limited the iterations to 100 to prevent long runtimes of the algorithm.

The histogram-based outlier detection calculates lower and upper thresholds of the segmented point cloud for the x-, y-, and z-components as well as for the diagonals of the xy-plane. Subsequently, to ensure height sensitivity, this procedure was repeated for three-meter-high slices of the point cloud. The thresholds were calculated to never exclude more than 20% of the considered point cloud to prevent removing parts of the tree of interest. This approach effectively reduces the size of the point cloud bounding box by removing small, detached branches belonging to neighboring trees and other artifacts without cutting into the actual tree of interest. However, this approach may result in some small branch tips being excluded from the dataset, as they might be incorrectly segmented as part of neighboring trees. These missing parts are typically on the outer edges of the tree crowns and do not significantly affect the overall structure or dimensions of the tree.

The code of this algorithm is available in the dataset’s GitLab repository (https://gitlab.gwdg.de/griese1/biodiv-3dtrees).

Finally, we split the point clouds into their respective ULS and TLS parts and saved them separately. This workflow ensures that TLS and ULS segments are produced through identical processing steps, leveraging the higher resolution and data quality of the TLS data to enhance the segmentation of both datasets.

The tree species labels were added based on an existing forest inventory dataset of the Biodiversity Exploratories^20^. For this, each segmented point cloud had to be assigned to a tree from the forest inventory dataset. To this end, the TLS-based stem position of the cleaned segments was extracted using the median XY-coordinates of a 20 cm cut at stem base level. Since the tree positions of the forest inventory did not directly align with the point cloud–based tree positions, a spatial transformation was required to enable accurate matching. Therefore, we transformed the point cloud-based positions to match the forest inventory dataset using extended coherent point drift as implemented in the Python package *probreg* (version 0.3.8)^30,31^. Finally, each TLS-based stem was assigned to the closest corresponding tree in the forest inventory. Assignments were only retained if the distance between the TLS-based stem and the inventory entry was less than or equal to 2 meters. This method resulted in a mean matching distance of 0.22 m. With that, tree species labels and a reference DBH could be used for further steps.

Using the R package *ITSMe* (version 1.0.0)^32^ we derived the TLS-based diameter at breast height (DBH) of each tree. To measure at the correct height of 1.3 m above ground, a digital terrain model (DTM) based on the TLS data was created and provided to *ITSMe*. Using the ULS point clouds, we derived the tree height relative to the corresponding ground level taken from the TLS-based DTM.

#### Tree selection

The size of the dataset made a full manual validation of the segmentation and matching to the forest inventory data without pre-filtering unfeasible. Therefore, we used the DBH measurements of the forest inventory dataset as well as the commonly assumed general relationship between DBH and tree height to remove unrealistically dimensioned and mismatched tree segments. This was followed by a manual validation of the tree segmentation.

We only kept segments for which the following three conditions hold, ensuring high-quality data:

1. *Maximum:* Point cloud-based DBH is not bigger than the sum of the maximum DBH of the preceding forest inventory across all plots, the expected maximum diameter increment based on the German National Forest Inventory (NFI)^33^, and the assumed TLS measurement error of 5 cm.
2. *Concrete Tree:* The absolute difference between forest inventory-based diameter and the point cloud-based diameter is not bigger than the sum of the assumed TLS measurement error of 5 cm and the expected diameter increment based on the German NFI.
3. *Minimum:* Point cloud-based DBH is at least 7 cm, following the German NFI^33^.

The expected maximum diameter increment was calculated from the average expected basal area increment across all tree species as reported by the German National Forest Inventory 2022^33^. We used a measurement error of 5 cm to account for the TLS device inaccuracy, the circle fitting error, as well as potential stem obstructions by e.g., climbing plants like ivy (*Hedera helix*). Condition 2 was used to remove mismatched trees, as well as trees with bad point cloud coverage and therefore inaccurate TLS-based DBH. Condition 3 was used to remove tiny trees, whose point clouds were often also of poor overall quality.

Assuming that the relationship between the derived point cloud-based DBH and tree height follows the well-known sigmoidal curve, we fitted the model used previously^34^:

$$H=a\cdot {\left((,1-{e}^{-b\cdot {DBH}},)\right)}^{c}\,.$$

**Fig. 2:**
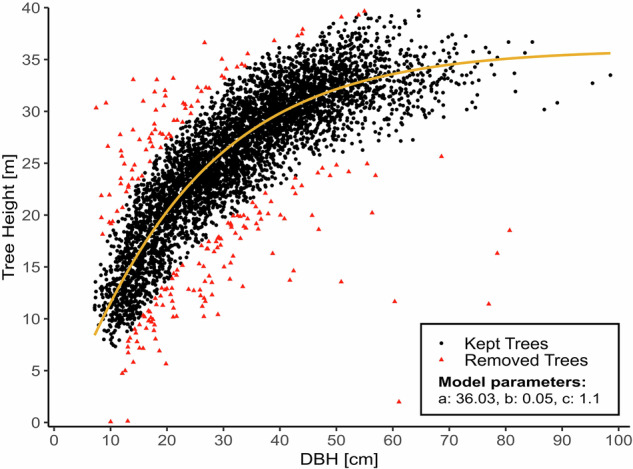
Scatter plot of the relationship of DBH and tree height derived from the single tree segments. Points in red mark rejected segments outside the defined 2σ interval of the fitted sigmoidal model (orange line).

Where *H* is the tree height in meters, *DBH* is the diameter at breast height in cm, and *a*, *b* and *c* are the model parameters. We then defined outliers using double the standard deviation (2σ) of the residuals of the model, which were rejected as final step of the selection pipeline (see Fig. 2). This step removed 3.9% of the data.

**Fig. 3:**
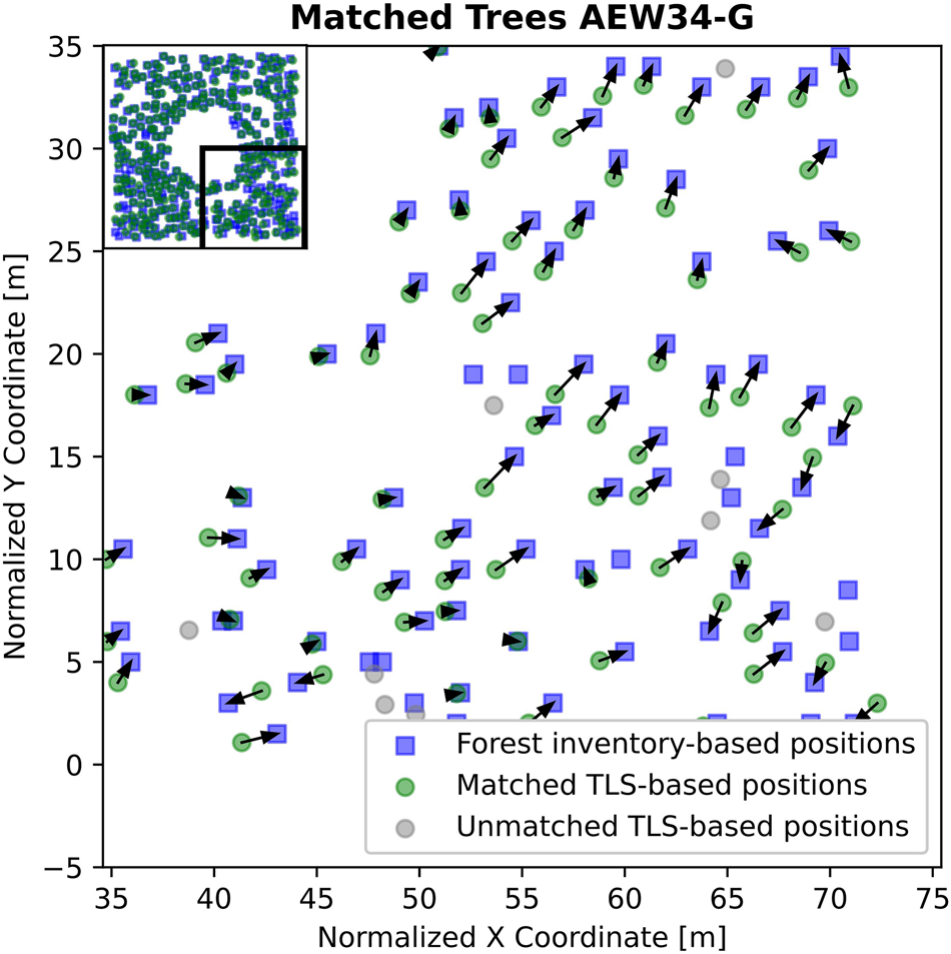
Example of the matching of TLS stem positions and the forest inventory data on a part of AEW47-G. Points in gray mark unmatched TLS-based stem positions, while point in green mark successfully matched ones. Blue squares mark the stem positions as taken from the forest inventory. The subfigure in the top left corner shows the position of the magnified part of the plot.

After this selection process, we were left with 5,142 trees in total. An example of the matching results can be seen in Fig. 3.

#### QSM reconstructions

Since the quality of QSM reconstructions is dependent on the quality and completeness of woody parts depicted in the input point cloud^35^ and coniferous trees are often affected by a high amount of occlusion caused by the needles^36,37^, we only used point clouds of the 3,452 broadleafed trees for reconstruction. We used TreeQSM (version 2.4.1) as reconstruction algorithm as it is one of the most widely used and validated algorithms and its script-style allowed efficient batch processing^35^. Before reconstruction, noise points were removed using the statistical outlier removal filter (n = 10, k = 2) as implemented in the Python package *open3d* (version 0.19.0)^38^. In TreeQSM, we used the function *define_input* to generate a grid of 16 parameter sets defining an internal preprocessing step during reconstruction, following the standard procedure of applying TreeQSM. For each parameter set, 5 QSMs were derived based on different random initializations. We selected the best-fitting QSM for each tree based on the smallest average point-cylinder distance using TreeQSM’s *select_optimum* function. We then saved the selected ‘optimal’ QSM with the suffix ‘raw’, as well as all reconstruction versions of each tree with the suffix ‘all’ as MAT files, which can be found as part of the dataset.

To correct for overestimated branch diameters, we applied the allometric correction implemented in the R package *rTwig* (version 1.4.0)^39^. The correction model uses the average twig diameter in millimeters as its only parameter and provides a database of 104 twig diameters of different species and genera. Destructive validation showed that *rTwig* enhances the biomass estimation by reducing the relative mean error to −1.2% and the relative root mean square error to 10.5%^39^. We used the previously assigned tree species as input for the allometric correction. If a species was not included in the database of *rTwig*, we resorted to the parameter valid for the genus of the tree. In case of European Hornbeam (*Carpinus betulus*), we used the twig diameter of American hophornbeam (*Ostrya virginiana*) due to their phenological similarity. The corrected QSMs were saved as CSV files which served as input to the graph reconstruction and cleanup described next.

#### Graph Representation

The graph representation builds on the *parent_id* from TreeQSM which for every cylinder refers to its parent. In some cases, especially for twigs and in areas of low point density, TreeQSM produced unrealistic connections between cylinders, sometimes even connecting cylinders on opposite sides of the tree. To get a proper graph structure from the QSM, we performed excessive cleanup on the dataset to remove these artifacts and ensure more accurate connectivity. The cleanup implements a heuristic for the parent-child relation, assessing the spatial validity of each connection made by TreeQSM. We discard connections where the distance between the end of the parent cylinder and the start of the child cylinder is too large relative to the parent’s radius. Central to this process is the requirement that connections align toward the trunk, ensuring the tree’s natural structure is preserved. To locate the best-fitting parent cylinder, we looked for cylinders in the vicinity while prioritizing connections where the angle between the fitted cylinder and the updated cylinder was small. If there was no potential parent cylinder that we could connect to directly, i.e. slightly modifying the predicted cylinder to improve the tree structure, we introduced an additional edge connecting the child cylinder to the best-fitting parent. This effectively introduces another cylinder into the model that only exists in the graph data because it is not supported by the point cloud directly, but rather by the structural requirement that branches do not sprout out of thin air. Based on the cleaned-up data, we recomputed all other fields, except for the cylinder geometries, in the csv files from *rTwig*. The updated CSV files of each tree are the final QSM representations with the suffix ‘cor’ in the dataset. The cleaned-up graph is additionally stored as GraphML files for convenient use in Python’s *networkx* package^40^.

## Data Records

**Fig. 4:**
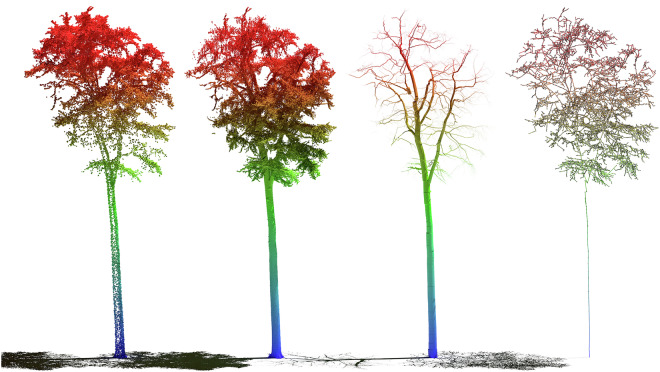
Visualization of the four data types contained in the dataset using tree HEW29_GD_174. From left to right: ULS point cloud, TLS point cloud, QSM, Graph.

**Fig. 5:**
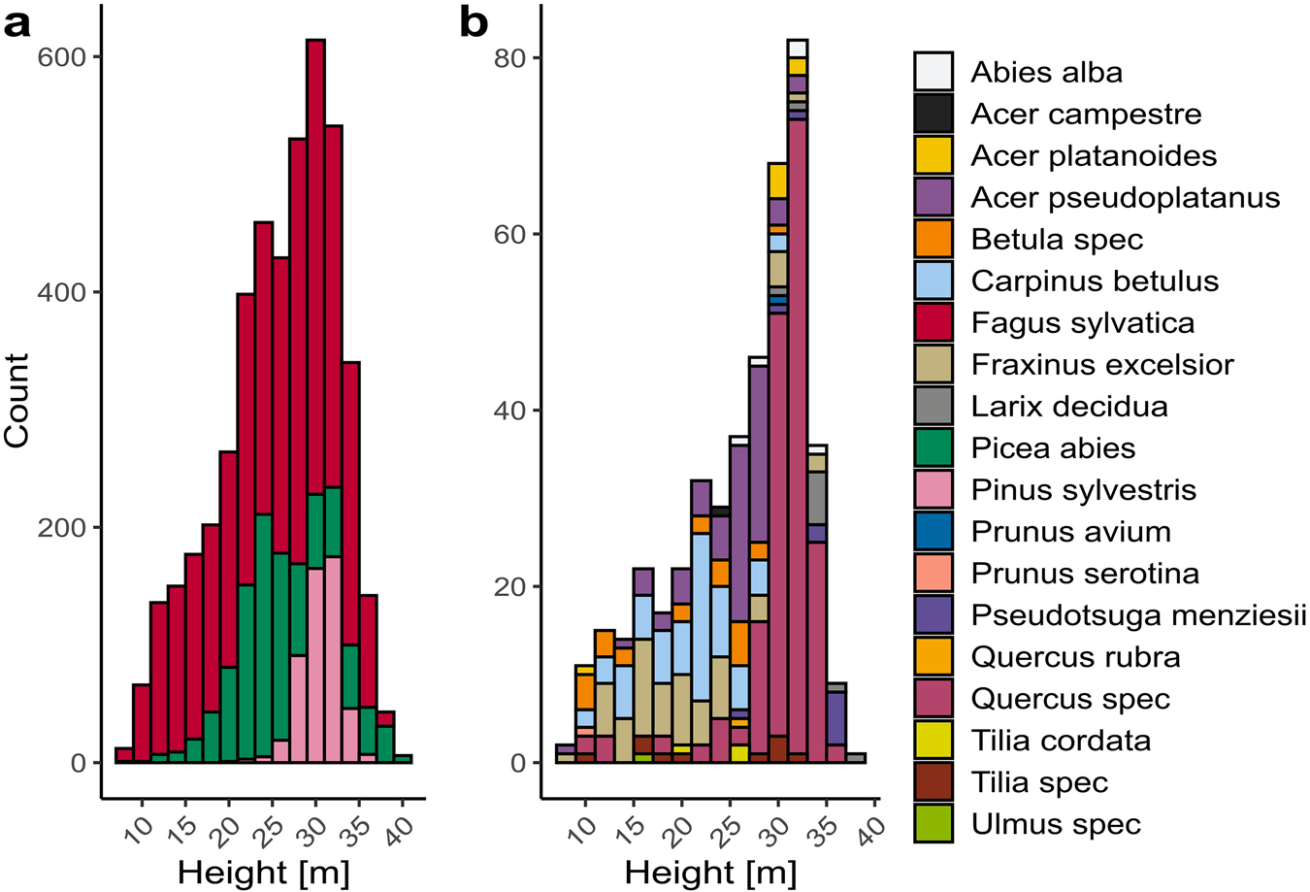
Tree height distribution of the trees in the dataset. The three main tree species *Fagus sylvatica*, *Picea abies*, and *Pinus sylvestris* are shown in (**a**), whereas the other species are shown in (**b**).

The dataset is available to download under GROdata^19^. It comprises TLS and ULS point clouds of the segmented single trees, as well as the QSMs of the broadleafed trees and their respective graph representations (see Fig. 4). The most frequent tree species in BioDiv-3DTrees are European beech, Norway spruce and Scots pine with diameters ranging from 7 cm to 98 cm and tree heights ranging from 7.3 m to 39.7 m. These species account for 3,997 individuals (80.7%) of the dataset. The other 16 tree species have diameters ranging from 7 cm to 89 cm and tree heights ranging from 8.9 m to 37.5 m (see Fig. 5).

Each part of the dataset is in a designated folder named after their data type (TLS, ULS, QSM, Graph). The dataset includes pre-defined training (80%), validation (10%), and test (10%) splits, generated using the *train_test_split* function of scikit-learn (version 1.6.1) with stratification by tree species^41^. The split labels can be found in the column *split* in the *‘labels.csv’*.

The *‘labels.csv’* also includes tree species labels, single tree variables used in the process of creating this dataset, the QSM-based tree volume in m³, as well as information whether the tree was selected for QSM reconstruction and passed the QSM validation steps.

Both TLS and ULS point clouds are provided as compressed LAZ files. The allometrically and topologically corrected QSMs are provided as CSV files following the standardized Real Twig format^42^, while the raw QSMs are provided as MAT files. For the CSV files, each row corresponds to a cylinder defined by its axis starting coordinate (*start_x, start_y, start_z*), the cylinder’s axis direction as the unit vector (*axis_x, axis_y, axis_z*) and the length and radius of the cylinder (*length*, *radius*). All coordinates in the dataset were normalized using the official gap center coordinates of the Biodiversity Exploratories^43^. If needed, absolute coordinates of the trees can be restored by adding the corresponding gap center coordinate to the point coordinates of each tree. An example of that can be found in the *getAbsoluteCoordinates.R* script provided in our GitLab repository (https://gitlab.gwdg.de/griese1/biodiv-3dtrees/).

All files are named after the same scheme based on the FOX-plots identifier, the tree number given by TreeLearn, and the data type separated by underscore characters (e.g. AEW03_G_101_TLS.laz is the TLS point cloud of the 101st tree on FOX-plot AEW03-G).

## Technical Validation

**Fig. 6:**
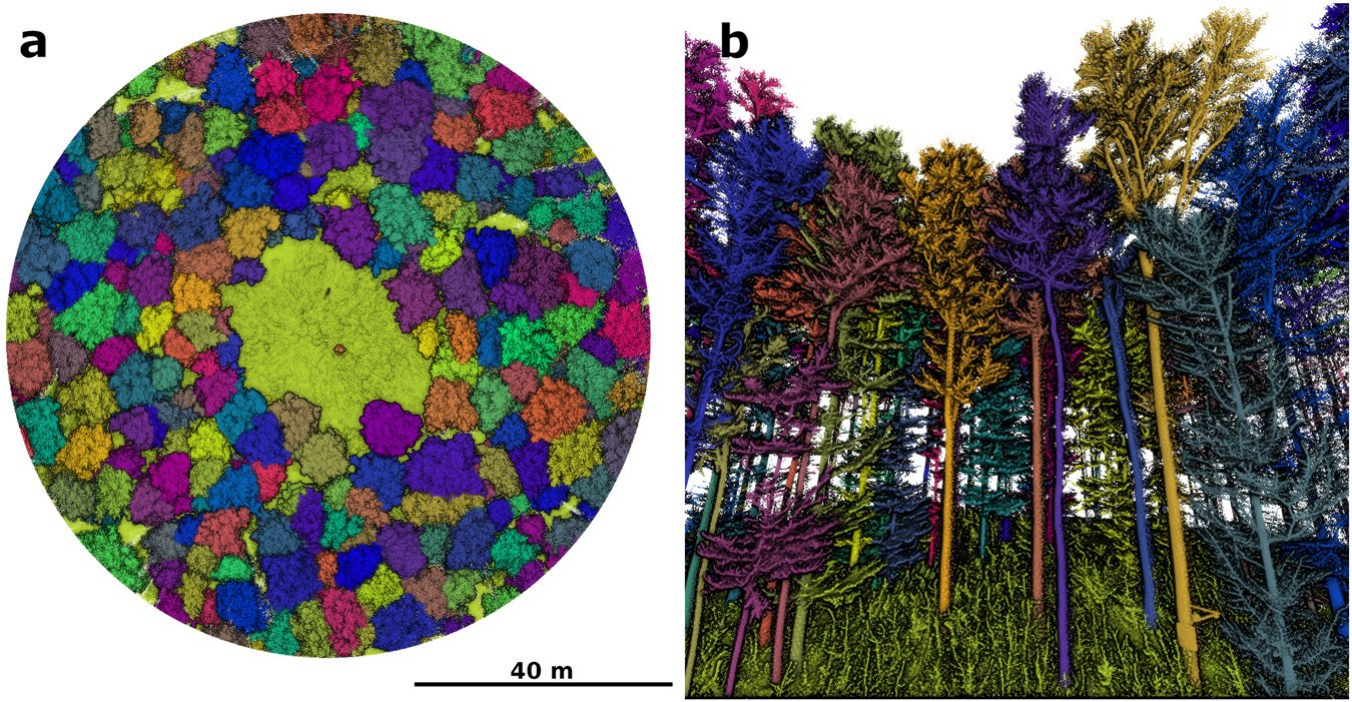
Accepted segmentation results at the example of HEW05-G. *a* shows an overview of the experimental plot with color-coded segmentation IDs. *b* shows an internal view from the gap center towards the forest, illustrating the quality of segmentation at branch and trunk level.

We manually validated the segmentation using plots of the XY-, XZ- and YZ-projections of the point clouds of each tree. If no over- or under-segmentation was visible, a tree was considered correctly segmented. In Fig. 6, an example of trees considered correctly segmented can be seen. After this step, 190 trees were removed, resulting in a final dataset size of 4,952 trees.

To be able to validate the QSMs we derived tree height, DBH, and crown projection area (CPA) from both the QSMs and TLS point clouds and compared the results. We assumed that if the reconstruction was successful and complete, there should not be a notable difference between QSM-based and TLS-based metrics.

**Fig. 7:**
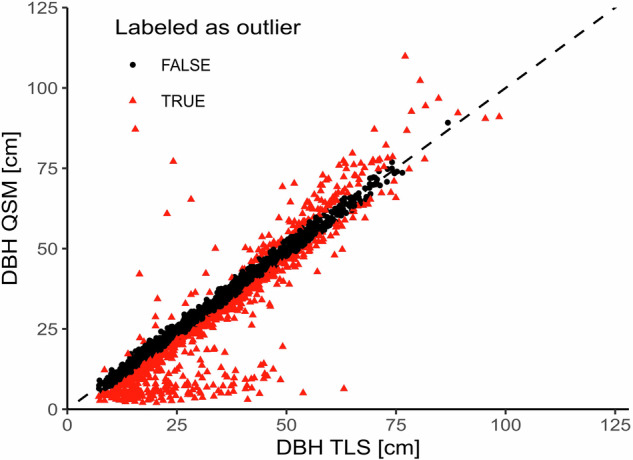
Scatter plot comparing the DBH of the derived QSMs with the DBH of the underlying point clouds. Points in red are outside the defined ±2 cm interval and were labeled as outliers (864 trees), while black points are inliers (2,615 trees).

**Fig. 8:**
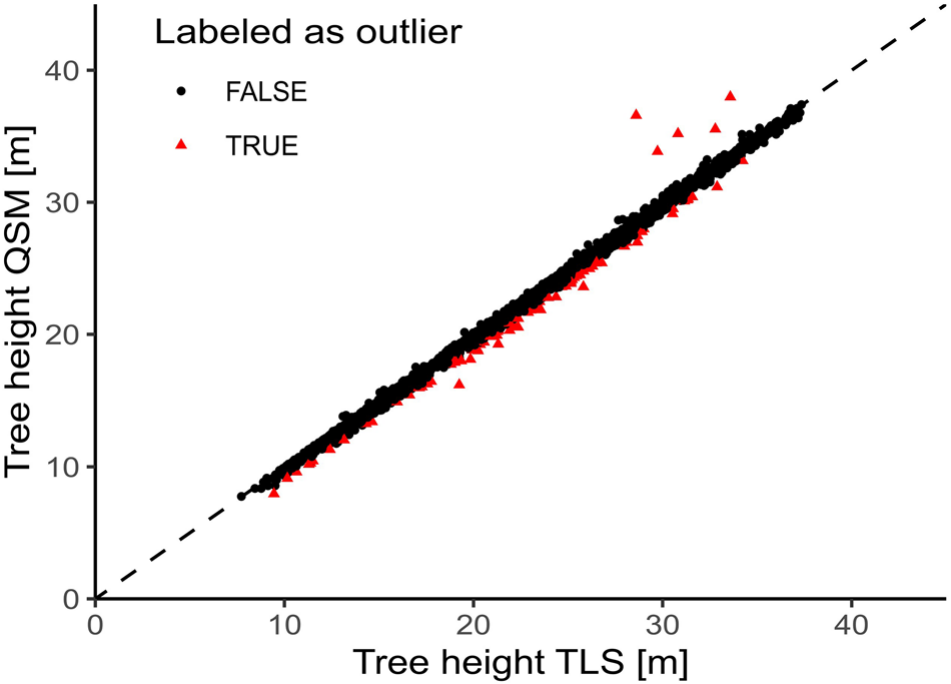
Scatter plot comparing the Tree height of the derived QSMs with the Tree height of the underlying point clouds. Points in red are outside the defined ±1 m interval and were labeled as outliers (95 trees), while black points are inliers (3,384 trees).

**Fig. 9:**
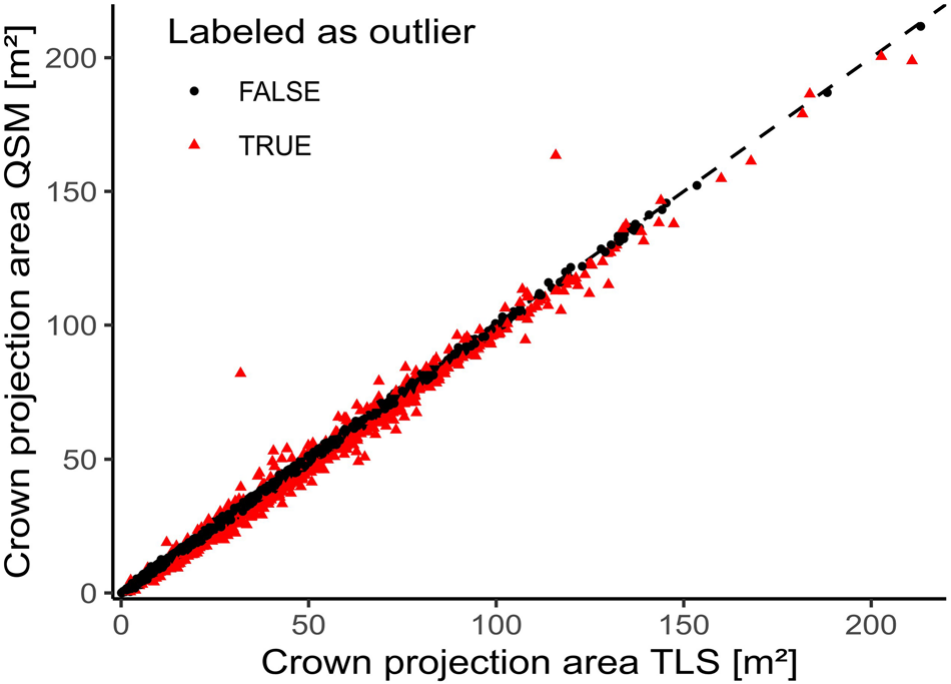
Scatter plot comparing the Crown Projection Area of the derived QSMs with the Crown Projection Area of the underlying point clouds. Points in red are outside the defined ±2 m² interval and were labeled as outliers (874 trees), while black points are inliers (2,605 trees).

For calculating the point cloud-based DBH and tree height we used the R package *ITSMe* (version 1.0.0)^32^ and for the QSM-based DBH and tree height we used the R package *rTwig* (version 1.4.0)^39^. Since the definition of CPA differs between the two packages, we derived CPA as the area of a concave hull of the 2D-projection of the point cloud and the QSM (concavity = 2) using the implementation in the R package *lidR*^44,45^. Using empirically chosen thresholds around the identity line (y = x), we classified outliers. Trees within the defined interval were considered valid. We expected that the DBH of the QSMs should fall within ±2 cm of the point cloud measurement, the tree height within ±1 m, and the CPA within ±2 m² of the point cloud-based value to be classified as valid reconstruction. Four Boolean columns *validDBH*, *validH*, *validCPA*, and *validQSM* were added to ‘*labels.csv’* to mark the different validation steps. The Boolean column *validQSM* is True if all preceding validation steps were passed, which was the case for 1,925 trees. Figures 7, 8, and 9 show the scatter plots comparing the QSM-based tree metrics and their point cloud-based equivalent.

## Usage Notes

Since ULS data is limited by factors like scanner capabilities and setup, flight speed and height, and vertical structure of the scanned forest, there are cases where trees are only fully visible in the TLS data. This is mostly the case for small trees of lower layers, which are occluded by overhanging neighboring trees. To be able to select only trees, which are visible in both data types, we added the number of points of the ULS point clouds as column *nULS* in the ‘*labels.csv’*.

## Data Availability

The complete dataset is available on GROdata (10.25625/8PB1IF).

## Code availability

The code of the used histogram-based outlier removal algorithm, as well as the code to reverse the coordinate normalization is available at the dataset’s GitLab repository (https://gitlab.gwdg.de/griese1/biodiv-3dtrees/). The repository also includes the code that creates and cleans up the graph representation.
